# United States Physician Preferences Regarding Healthcare Financing Options: A Multistate Survey

**DOI:** 10.3390/pharmacy6040131

**Published:** 2018-12-09

**Authors:** Shamima Khan, Joshua J. Spooner, Harlan E. Spotts

**Affiliations:** 1 Leon Hess Business School, Monmouth University, 400 Cedar Avenue, West, Long Branch, NJ 07764, USA; 2 CRE Services, Inc., 1560 Broadway, Suite 812, New York, NY 10036, USA; 3 College of Pharmacy and Health Sciences, Western New England University, 1215 Wilbraham Road, Springfield, MA 01119, USA; jspooner@wne.edu; 4 College of Business, Western New England University, 1215 Wilbraham Road, Springfield, MA 01119, USA; hspotts@wne.edu

**Keywords:** healthcare financing, physician survey

## Abstract

**Background:** Not much is currently known about United States (US) physicians’ opinions about healthcare financing, specifically subsequent to the creation and implementation of the Affordable Care Act (ACA). **Objectives:** A four state survey of practicing US based physicians’ opinions about healthcare financing following ACA passage and implementation. **Methods:** Physician leaders practicing in the state of New York, Texas, Colorado and Mississippi were surveyed. Two factor analyses (FA) were conducted to understand the underlying constructs. **Results:** We determined the final response rate to be 26.7% after adjusting it for a variety of factors. Most physicians favored either a single payer system (43.8%) or individualized insurance coverage using health savings accounts (33.2%). For the single-payer system, FA revealed two underlying constructs: System orientation (how the physicians perceived the impact on the healthcare system or patients) and individual orientation (how the physicians perceived the impact on individual physicians). Subsequently, we found that physicians who were perceived neutral in their attitudes towards physician-patient relationship and patient conflict were also neutral in reference to system orientation and individual orientation. Physicians who were perceived as stronger on the physician-patient relationship were more supportive of a single-payer system. **Conclusion:** This study brings attention to the paradox of social responsibility (to provide quality healthcare) and professional autonomy (the potential impact of a healthcare financing structure to negatively affect income and workload). Efforts to further reform healthcare financing and delivery in the US may encounter resistance from healthcare providers (physicians, mid-level prescribers, pharmacists, or nurses) if the proposed reform interferes with their professional autonomy.

## 1. Introduction

The run up to the creation of Affordable Care Act (ACA) in March 2010 furthered the ongoing debate regarding the ideal structure of healthcare financing in the United States [1,2,3]. Healthcare financing options span a spectrum of ideas, from a tax-payer funded unique payment option which includes universal coverage (also known as single payer system with universal coverage) on one end and a freely competitive market on the other; along this spectrum lie government-supported programs such as Medicare and Medicaid, employer sponsored health insurance plans, and individual insurance funded through health savings accounts. The suitability and viability of every option on the spectrum of health coverage generates passionate debate amongst health policy analysts, payer organizations, healthcare practitioners, and patients regarding social responsibility versus personal responsibility for care [4,5,6]. The structuring of healthcare financing in the United States stands in contrast to the framework of healthcare in industrialized nations such as Japan, Australia, Canada, and most of Europe, where universal coverage is mandated through legislation and funded primarily through taxation [7]. Within this framework, significant differences exist by which countries offer their universal coverage (including policies, service coverage, and copayment levels) [8]. For example, Switzerland’s policy of requiring residents to purchase compulsory basic health insurance with the option of purchasing supplemental private insurance contrasts with Norway’s program of taxation-based financing with equal access and coverage for all [9]. Despite these fundamental differences, Switzerland and Norway are both ranked in the top five of recent national consumer health indices [10].

Historically, the delivery mechanism for healthcare in the industrialized world followed the fee-for-service model, a system in which a patient’s clinical interest in receiving as much care as necessary was aligned with a physician’s financial interests [11]. This fee-for-service model has evolved to include a mix of capitation and fee-for-service systems, with financial incentives to ensure and advance the value of care [12]. The implementation of managed care cost control principles (including capitation, utilization review, physician profiling, and fee reduction) served to limit physician autonomy and curtailed American physician reimbursement [13], slowing healthcare expenditures for a period of time [14] but also increasing physician dissatisfaction with the American healthcare system and impinging their professional autonomy [15,16]. Physician satisfaction in the United States lags behind most industrialized nations, and is significantly lower than that in Switzerland and Norway [17]. Physician satisfaction is often tied to feelings of professional autonomy, the degree of bureaucratic interference, and payment rates [18].

Studies have been performed to determine the views of American physicians on healthcare financing options [19,20,21,22,23,24,25,26]; several of which [22,24,25] were published during the debate around the ACA. While these studies have found increasing physician support for a publicly financed single payer system, most were state or regional studies that were limited in scope. One national survey [22] found physicians were four to five times as likely to express support for either individually purchased insurance coverage with tax incentives and penalties or a government-run, taxpayer-financed national health insurance program compared to the current employer-sponsored financing system. To update this previous research and in order to explore for changes in opinions following the enactment of most of the key provisions of the ACA in January 2014, the authors conducted a four-state survey of practicing physicians.

## 2. Methods

### 2.1. Study Sample

Physician leaders practicing in the states of New York, Texas, Colorado, and Mississippi were selected for survey inclusion because of certain unique attributes. Texas had the highest rate of uninsured residents (28.8%) in 2012, far exceeding the national average of 16.9% [27]. Mississippi had a lower rate of uninsured residents (21.7%), but also had the greatest physician shortage in the country [28]. In contrast to Mississippi, New York state had the third highest concentration of practicing physicians in the nation (277.4 per 100,000 people). Nationally, in addition to the lack of physicians, Mississippi had the highest prevalence of diabetes (15.4%) and second highest prevalence of hypertension (38.4%). Colorado stands in stark contrast to Mississippi; the prevalence of hypertension (22.0%) and diabetes (7.4%) among Coloradans were the lowest and second lowest in the nation, respectively; Colorado also had the lowest rates of obesity nationwide [29].

The lead physician in the respective practice(s) were the focus of this survey. A third party, commercial vendor was employed to generate contact information for a practice’s lead physician; this vendor had no relationships with any of the included states’ medical societies. The selected vendor was utilized because they had the capability to provide lead physician contact information, which was not available from the respective state medical societies mailing lists. The study was funded by the College of Pharmacy and Health Sciences and approved by each author’s Institutional Review Board (IRB). 

The initial phase of this mixed method (paper, internet, fax, and telephone) study had each lead physician receive a packet which included a cover letter, survey instrument, and a postage-paid return envelope. A random sample of Internal Medicine and Family Practice physicians (total number of physicians = 2225) were selected for the initial mailing, distributed as follows: Colorado = 228, Mississippi = 153, New York = 893, and Texas = 951. Signed informed consent letters were not required, as a statement in the cover letter implied consent upon the return of a completed survey. Non-responding physicians received up to three reminder post cards and up to three additional mail surveys. Data collection for the study commenced in March 2014.

Additional attempts were made to increase the response rate by the Principal Investigator (PI) by contacting non-responding physicians via telephone using an institutional polling center at the authors’ institutions. A phone survey was conducted for those physicians indicating a preference for this survey method. Physicians also had the option to complete the survey online; an electronic copy was sent over the internet to those indicating this preference.

### 2.2. Survey Instrument Development

The survey instruments were developed after conducting a comprehensive literature review [19,20,22,30,31,32,33,34,35,36,37,38,39,40].

The survey was organized into four sections, including questions relating to: Various elements of the Patient Centered Medical Home (PCMH); patient-practitioner orientation; healthcare finance; and demographics. Close-ended, multiple choice questions were structured using dichotomous, and, 3-point and 4-point Likert type scales questions. 

The survey included 19 questions in reference to physician opinions about four different models of healthcare financing: A taxpayer-financed, national health insurance program (“single payer with universal coverage”) administered by the government, employer-sponsored health insurance, individually purchased insurance coverage paid for through health savings accounts, or a multi-payer managed care system (with federal subsidies available for low income subscribers and penalties for failure to purchase a minimum policy). The first eight questions determined physician opinions regarding the various healthcare financing options and attitudes towards healthcare access. These questions were adopted from a survey instrument developed by Albers et al. and McCormick et al. [19,20]. The original survey instrument developed by Albers et al. included eight questions excluding demographic questions (19). The remaining nine questions determined physician beliefs about the impact of a potential single payer system; these questions were adopted from a larger 50-item questionnaire developed by Nayakama et al. [35].

The 18-question Patient-Practitioner Orientation Scale (PPOS) developed in 2000 by Krupat was used as the basis of our measurement of patient-practitioner orientation [38]. A subset of seven questions from the PPOS focusing on the respondents’ philosophical belief regarding patients, physicians, and medical care were selected for use in our survey. An additional question was added regarding transparency of a physician’s relationships with the pharmaceutical and medical device industries [36]. A 4-point Likert scale was used (4: Strongly agree, 3: Agree, 2: Disagree, 1: Strongly disagree). Reverse wording was used for three questions (please see results section for detail); for these, the scale was reversed (with the lower scores associated with stronger agreement). The scale range for the patient-practitioner orientation measurement was from 8 to 32; the higher total score, the less patient orientation indicated by the physician.

### 2.3. Data and Statistical Analyses

Initial analyses included descriptive statistics (frequency counts, percentages, means, and standard deviations) for demographic and outcomes data, and other variables of interest. Relationships between physician characteristics and variables of interest were explored with additional analyses, including Chi-square [x^2^] analyses, regression and parametric statistics. Physician perceptions on the four different structures of healthcare financing was the primary outcome variable. Significance of the results was determined with an a priori *p* value of 0.05 or less. Statistical analyses were conducted using Statistix version 8 (Analytical Software, Tallahassee, FL, USA) and SPSS version 24 (Armonk, New York, NY, USA) [41,42].

To our knowledge, no previous research has investigated the interplay between patient-practitioner orientation and physician opinion on financing of the healthcare system. Survey responses regarding patient-practitioner orientation and perceptions on healthcare finance (including perceptions on the four different options for healthcare financing and a potential single payer system) were further analyzed. Two factor analyses using Principal Component Analyses (PCA) with Varimax rotations were performed to identify the components underlying the survey questions related to patient-practitioner orientation and perceptions about a single payer system. The objective of factor analysis is to take a large set of variables and examine how the data could be reduced to smaller set of factors or components [43]. Two sets of Chi-square analyses were conducted to explore potential relationships between the components obtained from the factor analysis of patient-practitioner orientation and (a) the four options of healthcare financing, and (b) components obtained from the factor analysis of physician perceptions of a potential single payer system.

## 3. Results

### 3.1. Survey Responses

**Figure 1:**
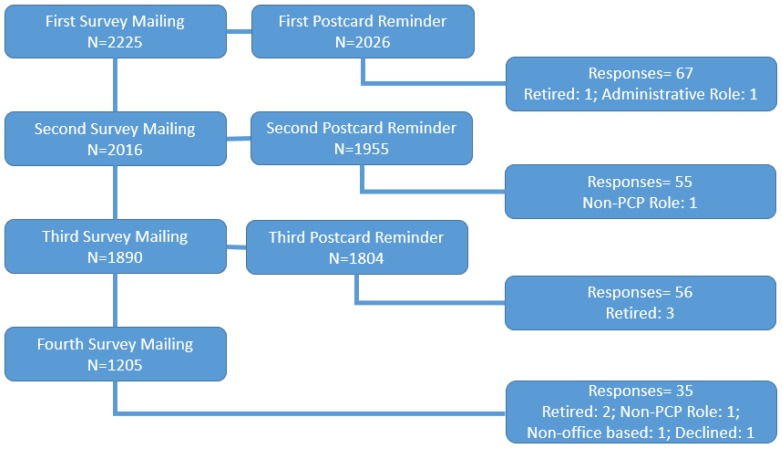
Mailing of surveys.

**Figure 2:**
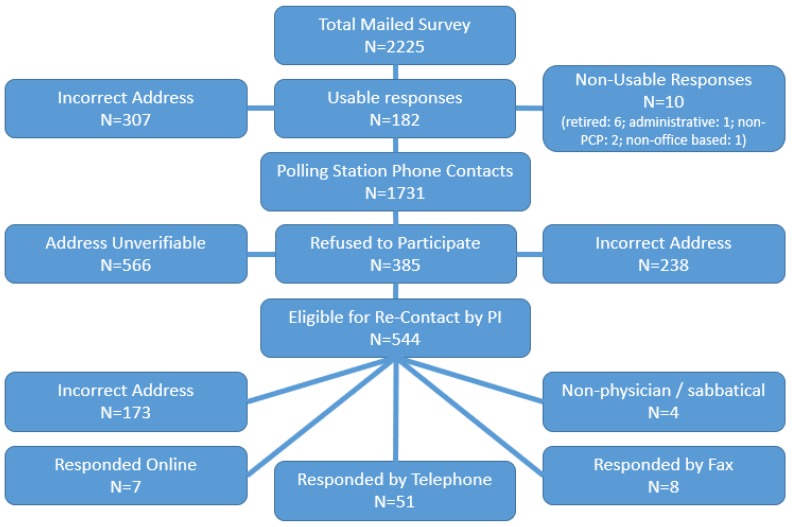
Polling station contacts.

**Figure 3:**
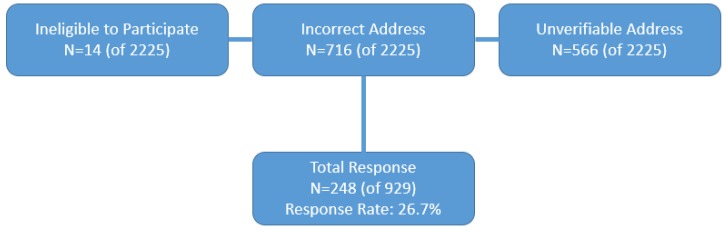
Survey response rate.

The initial mail survey of 2225 lead physicians resulted in 182 useable responses, with 307 surveys returned as undeliverable. After repeated mailings, attempts to increase response rates were made by contacting all non-responding physicians (*n* = 1731) during the summer of 2015 via telephone by a university-based polling station. An additional 802 physicians were eliminated from the sample for the following reasons: Incorrect addresses (*n* = 236), unverifiable addresses (*n* = 566), or refusal to participate (*n* = 385). The remaining 544 non-responding physicians were then re-contacted by the PI; an additional 173 addresses were deemed incorrect. These efforts to reduce non-response yielded an additional 66 survey responses (51 via telephone, 8 via fax, and 7 via online survey). This resulted in a total of 248 responding physicians, representing a final response rate of 11.1% and an adjusted response rate of 26.7% (after correcting for incorrect and unverifiable addresses) (Figure 1, Figure 2 and Figure 3).

### 3.2. Baseline Cohort Description

Responding physicians worked in small practices, with solo practices constituting half (50.2%) the sample; most of the sample (81.8%) reported working in practices with five or fewer physicians (Table 1). State medical association membership was reported by most responding physicians (67.4%); commercial insurance and Medicare (33% each) were most frequently reported for patient coverage, and almost 8% of patients were reported as uninsured. Responding physicians correlated well with national physician demographic data in terms of age, gender, and membership in the American Medical Association [44,45]. There was some state-wise statistical variation on certain demographics: Patient insurance coverage mix (*p* < 0.05), state medical society membership (*p* < 0.0001), and rural/suburban/urban location (*p* = 0.0214).

### 3.3. Healthcare Financing Options

Table 2 reports information about the four different healthcare financing options in reference to respondent demographic information. Of the four options, most physicians favored either a single payer system (43.8%) or individualized insurance coverage using health savings accounts (33.2%). It is worth noting that neither of these systems are currently utilized to finance the United States healthcare system; the financing systems currently most frequently utilized in the United States were least favored by the respondents: Multi-payer managed care (17.7%) and an employer sponsored healthcare system with tax credits or penalties (5.3%). Only a minority of demographic data provided statistically significant differences: Physicians who were not members of their state medical society were more likely to prefer a tax-payer funded unique system with universal coverage over individualized insurance with health savings accounts compared to members of state medical societies, who were equally likely to favor these two options (Table 2). Physicians practicing in Texas reported a slim preference for a tax-payer funded unique payment system with universal coverage compared to physicians practicing in other states (Table 2).

Table 3 represents the overall physician attitudes towards access to healthcare and a single payer system, and Table 4 identifies statistically significant differences between demographic variables. When physicians were asked about patient access to good medical care, a vast majority (86.3%) either strongly agreed or agreed that ‘good medical care should be accessible to all regardless of ability to pay.’ However, less than half (43.8%) favored a single payer system (Table 2), and even fewer (34.6%) favored a single payer system like England.

We found a number of demographic and practice-related factors that exerted influence on physician opinion about access to healthcare and a single payer system. Fewer state medical society members favored a unique payment mechanism (29.4% vs. 46.2%) and were more likely to favor the use of health savings accounts with high deductibles (43.2% vs. 32.1%) compared to those who reportedly were not state medical society members (Table 4). Further, more than half of non-members felt that fewer administrative staff would be necessary under a single payer system (65.3% vs. 39.1%, Table 4) and the overall US economy would benefit from such a system (56.6% vs. 39.4%) as compared to members (Table 4). Additionally, compared to physicians practicing in rural locations (46.8%), physicians practicing in urban locations (58.5%) were more likely to indicate that fewer administrative staff would be necessary under a single payer system (Table 4). More rural physicians (practicing in rural areas) favored the use of health savings accounts with a high deductible compared to those practicing in urban locations (70.5% vs. 55.8%) (Table 4). 

Physicians who disagreed or strongly disagreed with the statement that a unique payment mechanism requires fewer administrative staff were spending a higher percentage of their time in patient care (mean = 87.5% of time) compared to their peers whose opinion differed with that assertion (81.5% of time; *p* = 0.0143; data not shown). Patients’ Medicaid enrollment status influenced physician beliefs in several ways. Physicians who favored a single payer system, like England, had a higher percentage of their patients enrolled in Medicaid (mean = 21.4%) compared to those who were undecided or opposed to such a plan (14.2%, *p* = 0.0245; data not shown). The use of health savings accounts with high deductibles were less likely to be favored by physicians with higher mean percentages of their patients enrolled in Medicaid (12.6% vs. 21.1%; *p* = 0.0178, data not shown), while physicians who were more likely to strongly agree or agree that their income would decrease under a single payer system had a lower mean percentage of patients enrolled in Medicaid (13.2% vs. 20.8%; *p* = 0.0071). 

### 3.4. Patient-Practitioner Orientation

Responses to the patient-practitioner orientation questions were mixed (see Table 5); half the questions reflected agreement with patient orientation (85% or higher), but the remaining questions reflected less agreement with patient orientation (a response of 70% or less). For the statement, “most patients want to get in and out of the physician’s office as quickly as possible,” only 48.1% of respondents disagreed or strongly disagreed.

The patient-practitioner orientation measurement consisted of two subscales, one for Sharing (mean = 3.22) and the other Caring (mean = 3.32). Physicians tended to be more patient-centric in terms of Caring when they were not members of the American Medical Association (mean = 3.6 vs. 3.3, *p* = 0.005). Furthermore, for the Caring subscale, physicians located in New York tended to be more patient-centric versus physicians located in the other states (mean score 3.6 vs. 3.4, *p* = 0.04). 

### 3.5. Statistical Analysis: Healthcare Financing and Patient-Practitioner Orientation

A Factor Analysis (FA) of patient-practitioner orientation revealed three components, which explained about 52% of the variance. For patient-practitioner orientation, FA reduced the eight survey questions on patient-practitioner orientation to three components or factors. These three components, in order of importance, were determined to be: Physician-Patient Relationship (which explained 20.3% of the variance), Physician-Patient Engagement (18.0%), and Patient Conflict (13.6%) (Table 6). We found a statistically significant relationship between one factor (physician-patient relationship) and the four financing options (Table 7). Most physicians (67.4%) whose responses loaded strongly on the physician-patient relationship factor favored a single-payer system, compared to all other systems (each ≤ 18.6%) (Table 7). Conversely, physicians whose responses did not load strongly on the physician-patient relationship factor equally favored a multi-payer managed care system or individualized insurance coverage (using health savings account) (each 50.0%) and none favored a single-payer system. Respondents whose loadings were neutral mostly favored a single payer system (43.0%) or an individualized insurance coverage using health savings accounts (36.4%) (Table 7). 

The results of the FA for the single payer system revealed two components which explained 67.3% of the scale variance: System and individual orientations. System orientation (defined as how the physicians perceived the impact of the single payer system on the healthcare system or patients) was the dominant dimension, explaining 47.7% of the variance. Individual orientation (how the physicians perceived the impact of the single payer system on individual physicians) explained 19.6% of the variance (Table 8). We further examined the results of the FA by conducting a Chi-square analysis. The two factors of patient-practitioner orientation (physician-patient relationship and patient conflict) and both factors of the single payer system (Table 9) exerted statistically significant results. Respondents perceived neutral in their attitudes towards physician-patient relationship were also neutral in reference to system orientation (83.7%) and individual orientation (70.3%). Similarly, respondents perceived neutral in their attitudes towards patient conflict were also neutral in reference to system orientation (72.4%) and individual orientation (76.9%). Furthermore, physicians who were perceived to have weaker physician-patient relationships were less system oriented (16.7%) than those perceived to have stronger physician-patient relationships (0%). The opposite relationship was observed with physicians perceived to have stronger physician-patient relationships being more system-oriented (58.3%). A higher percentage (29.4%) of physicians who perceived less patient conflict were more system oriented than those physicians who perceived more patient conflict (4.7%). Finally, physicians who had perceived less patient conflict were less likely (44.1%) to have a neutral individual orientation than those physicians who had either perceived neutral (76.9%) or more (59.5%) patient conflict.

## 4. Discussion

In absence of a comprehensive nationwide study, this four state study provides us with significant information about physicians’ opinions about healthcare financing. Furthermore, the few studies that have examined physicians’ views about healthcare financing were conducted prior to the passage and implementation of the ACA [19,22,25]. Our most significant finding was an apparent contradiction regarding physician opinions about the financing system. Although a vast majority (86%) agreed that good medical care should be accessible to all regardless of ability to pay, only one-third (34.6%) favored a single payer system (similar to England) which would guarantee such access. This contradiction may be the product of physician concerns observed in our findings (and supported in previous research) that a single payer system would connect to a substantial decline in income and a rise in workload. Past research has found that physician backing for a single payer system is reduced if physician income is expected to be negatively impacted [35], eroding support for a system that physician respondents identified as having the potential to improve preventive care access and provide high quality emergency care. Nevertheless, it is worth noting that good medical care could be financed with a multi-payer system while providing universal coverage, a system which operates in Switzerland [46].

The percentage of respondent physicians (86%) that agreed that good medical care should be accessible to all regardless of ability to pay was aligned with older research (range: 86% to 89%), [19,22] indicating that physician views on this topic have not changed over time, despite the significant policy changes implemented by the ACA. One issue where physician perspective may have changed over time is the function of private insurance in the funding and delivery of healthcare; our study found 46.7% of respondents agreed or strongly agreed that private insurance should play a major role, higher than the 29.7% found in a previous study of Massachusetts physicians [20]. This difference may be explained in part by interstate variance in the survey population (statewide rates varied by 23.5%), or an endorsement of private health insurance by those respondents who are opposed to the ACA and/or a single payer system, which was conceptually closer to fruition at the time of our survey than the comparator survey.

Among the four healthcare financing options, respondent physicians most favored either a single payer system (43.8%) or individualized insurance coverage using health savings accounts (33.2%). The financing systems most frequently utilized in the United States (multi-payer managed care; employer sponsored healthcare system with tax credits or penalties) were not favored by physicians, with only one in twenty respondents indicating a preference for employer sponsored healthcare. These findings were consistently observed through our respondent pool, with few demographic characteristics contributing variance to the results. These findings are also similar to research conducted prior to the passage of the ACA, which found a high preference for a single payer system and low preference for managed care among surveyed physicians in Massachusetts [20] and Minnesota [19].

Geography may influence physician attitudes towards healthcare financing options, with Texas physicians less likely than other respondents to favor a single payer system with universal coverage. This result may reflect interstate differences more so than regional or political differences, as Texas and Mississippi are both heavily Republican states in the United States Census Bureau south central division [47]. Texas has both the highest rate and the highest number of uninsured individuals in the nation [27], despite having a median household income in the upper half among all states [48]. Despite being geographically and politically similar to Texas, Mississippi physicians were more in support of a single payer system with universal coverage. This may be a product of Mississippi having the lowest median household income in the nation [48]. There were no variations in our findings centered on practice location (urban/suburban/rural), in contrast to an earlier study [19].

Few other demographic variables exerted a statistically significant influence on physician attitudes towards healthcare financing options. Unlike previous research, our results found no significant influence of physician gender [19,20]. Membership in the American Medical Association was not associated with respondent opinions on healthcare financing, contradicting previous research [22]. However, physicians who were members of their state medical society were equally likely to prefer a single payer system or other individualized insurance with health savings accounts, whereas non-members of state medical societies were more likely to favor the single payer system. Though not clear, this finding may be the result of the higher percentage of membership by Texas physicians in their state medical society, relative to other states. Physicians who favored a single payer system had a higher percentage of their patients enrolled in Medicaid compared to those who were undecided or opposed to such a plan. Medicaid is associated with low reimbursement rates, delays in payment, and cumbersome paperwork requiring additional staff [49].

A 10% decrease in income in exchange for a substantial decrease in paperwork was agreeable to 61.5% of physicians, consistent to what was observed in previous research from the 1990s and 2000s [20,23,50]. This demonstrates that paperwork remains a significant burden to medical offices; this paperwork burden requires employment of administrative staff (such as medical billing clerks) and increasing operating costs. A plurality (53%) of our respondents agree (or strongly agree) that they would need fewer administrative staff under a single payer system. A single payer system is unlikely to lead to fewer patient visits or fewer services provided, which leads the authors to believe that the respondent’s need for fewer administrative staff is a product of either a reduced paperwork burden to the medical office (requiring fewer administrative staff to manage the paperwork) or a byproduct of decreased practice revenue/physician income (and the inability to afford administrative staff). Sixty one percent of our respondents agreed or strongly agreed that a single payer system would lead to a significant decrease in his/her income, a similar percentage was observed previously among American surgeons [35].

Finally, we believe that this is first article that has evaluated a relationship between patient-practitioner orientation and opinions regarding financing of the healthcare system. The results of factor analysis and subsequent Chi-square analyses revealed that physicians who were perceived as stronger on the physician-patient relationship leaned more towards a single-payer system, while physicians who were perceived as weaker in the physician-patient relationship did not favor a single payer system. Though a direct comparison from the literature is not feasible, previous research in this area has found that physicians do agree that a single payer system would advance patient access to preventive care and high-quality emergency care [35] which may connect with a stronger physician-patient relationship. Similarly, physicians who were perceived as stronger on the physician-patient relationship were more likely to favor a system-orientation. Lastly, the third factor capturing patient conflict appears to measure the level of trust in the physician-patient relationship. The more perceived conflict in the physician-patient relationship, the less likely the physician will favor either system or individual orientation towards a single payer system. It appears from this study that patient trust has a pivotal impact on the physicians’ mindset towards healthcare financing. 

Any substantial change in the way that healthcare is financed, including the methods examined in this study, would potentially impact physician income and the physician-patient relationship, but also many other facets of the US healthcare system, including pharmaceuticals. Given that, it is not advisable to speculate on the specific impact of these changes on pharmacy and pharmacists based on a survey of physician opinions. Consequently, the impact of a single payer system with universal coverage on pharmacy or pharmacists is conjecture at this point, and would not be fully elucidated until any reforms are developed and implemented. It is possible that prescription drug coverage under a single payer system could be installed as an extension of Medicare [51,52]. In that case, the prescription drug coverage could be an augmentation of the Medicare Part D coverage, which is currently available (as optional coverage) to all Medicare beneficiaries [51,52,53,54,55]. Medicare Part D as a method of drug coverage from the pharmacists’ and physicians’ perspective has been studied extensively [51,52,53,54,55].

## 5. Limitations

There are several limitations to this research, foremost the low survey response rate. A third party vendor (and not the customary medical society database or non-profit organization) was contracted to provide physician contact information and addresses [30,33,40,56,57]; inaccuracies within the vendor-provided list may have contributed to the low response rate. Furthermore, the targeting of lead physicians, who may be more time constrained with the additional leadership or managerial responsibilities of operating a primary care practice, may have led them to be less likely to respond to surveys compared to staff physicians. Nevertheless, our response rate is similar to a recent similar survey [40,58]. The survey did not collect information about physicians’ political beliefs, which may have influenced their outlook on healthcare financing. Modification of the variety of survey instruments utilized into one single survey was required to condense for size and increase the response rate [19,30,35,59]. Our questionnaire did not define the terms managed care and single payer system, similar to previous research [19,35]. Managed care encompasses various organizations and contracts that differ in their details, though the authors believe that physicians understand the fundamental relationships denoted by these terms. Lastly, similar to any survey, the results may be subject to a non-responder bias and social desirability response bias [53,54,55,56,57]. 

## 6. Conclusions

The results of this multistate study of physicians brings attention to the paradox of social responsibility (to provide quality healthcare) and professional autonomy (the potential impact of a healthcare financing structure to negatively affect income and workload). To garner more physician support, efforts towards restructuring the healthcare financing system should include preservation of physician income and workload issues.

## Figures and Tables

**Table 1:** Physician and practice characteristics.

Characteristics	Primary State of Medical Practice				Total
	NY (*n* = 112)	TX (*n* = 81)	CO (*n* = 13)	MS (*n* = 30)	
Male	82 (73.2%)	56 (69.1%)	12 (92.3%)	21 (70.0%)	175 (72%)
Geographical Location *					
Rural	31 (27.9%)	26 (32.1%)	8 (61.5%)	12 (40.0%)	78 (32.2%)
Suburban	29 (26.1%)	32 (39.5%)	4 (30.8%)	9 (30.0%)	75 (31.0%)
Urban	51 (45.9%)	23 (28.4%)	1 (7.7%)	9 (30.0%)	87 (36.0%)
Practice Size (# of Physicians Practicing)					
Solo	60 (55.0%)	49 (60.4%)	5 (38.5%)	8 (26.7%)	124 (50.2%)
2 to 5	27 (24.8%)	20 (18.3%)	4 (30.8%)	17 (56.7%)	70 (28.3%)
6 or more	22 (20.2%)	12 (14.8%)	4 (30.8%)	4 (13.3%)	45 (18.2%)
Member of State Medical Association **	54 (48.2%)	74 (91.3%)	9 (69.2%)	21 (72.4%)	163 (67.4%)
Member of American Medical Association	30 (26.8%)	22 (27.2%)	5 (41.7%)	6 (20.0%)	64 (26.4%)
Medical School Graduation Year					
Before 1970	11 (9.9%)	7 (8.8%)	0	2 (6.9%)	23 (9.3%)
1970 to 1989	69 (62.1%)	44 (55.0%)	13 (100%)	15 (51.7%)	143 (57.9%)
1990 to Present	31 (27.9%)	29 (36.3%)	0	12 (41.3%)	74 (30.0%)
% Patients Enrolled in Medicaid (mean ± std.dev) ***	19.6 (19.6)	11.3 (14.0)	25.3 (20.7)	16.5 (24.2)	16.5 (18.7)
% Patients Enrolled in Medicare (mean ± std.dev)	34.7 (21.4)	34.2 (21.5)	30.6 (17.9)	27.9 (21.6)	33.1 (21.4)
% Patients Enrolled in Commercial Insurance (mean ± std.dev)	29.5 (20.1)	35.3 (22.7)	27.6 (19.6)	40.8 (25.2)	33.3 (22.1)
% Patients Enrolled in HMO (mean ± std.dev) ****	15.8 (16.8)	9.6 (12.0)	3.8 (5.9)	11.4 (12.4)	12.3 (14.5)
% Patients Uninsured (mean ± std.dev) *****	6.7 (10.5)	8.7 (11.2)	15.0 (11.2)	6.2 (4.7)	7.8 (10.4)

Primary state of medical practice not provided by 12 respondents (4.8%). * x^2^ = 14.85; *p* = 0.0214; ** x^2^ = 40.17; *p* < 0.0001; *** Bartlett’s Test x^2^ = 14.7; *p* = 0.0021; **** Bartlett’s Test x^2^ = 18.6; *p* = 0.0003; ***** Bartlett’s Test x^2^ = 18.6; *p* = 0.003; Data presented as No (%)/Mean (± Standard Deviation (std.dev)).

**Table 2:** Four different healthcare financing options and demographic data.

	Multi-Payer Managed Care System	Single Payer with Universal Coverage	Individualized Insurance Coverage (Using Health Savings Account)	Employer Sponsored Healthcare System with Tax Credit or Penalty (For Buying or not Buying Health Insurance)
OVERALL	40 (17.7%)	99 (43.8%)	75 (33.2%)	12 (5.3%)
Gender				
Male	28 (17.4%)	71 (44.1%)	53 (32.9%)	9 (5.6%)
Female	11 (17.7%)	27 (43.5%)	21 (33.9%)	3 (4.8%)
State Medical Society Member				
Yes	25 (16.7%)	59 (39.3%)	59 (39.3%)	7 (4.7%)
No	14 (19.2%)	39 (53.4%)	15 (20.5%)	5 (6.8%) *
American Medical Association Member				
Yes	8 (13.8%)	28 (48.3%)	17 (29.3%)	5 (8.6%)
No	31 (18.9%)	70 (42.7%)	56 (34.1%)	7 (4.3%)
Medical School Graduation Year				
Before 1970	5 (23.8%)	7 (33.3%)	7 (33.33%)	2 (9.5%)
1970 to 1989	19 (14.4%)	64 (48.5%)	44 (33.3%)	5 (3.8%)
1990 or later	14 (20.3%)	27 (39.1%)	23 (33.3%)	5 (7.2%)
Geographic location of primary medical practice				
Rural	11 (15.1%)	36 (49.3%)	23 (31.5%)	3 (4.1%)
Suburban	14 (19.4%)	23 (31.9%)	28 (38.9%)	7 (9.7%)
Urban	14 (17.9%)	39 (50%)	23 (29.5%)	2 (2.6%)
Primary State of Medical Practice				
New York	24 (22.6%)	51 (48.1%)	24 (22.6%)	7 (6.6%)
Mississippi	0 (0%)	7 (53.8%)	6 (46.2%)	0 (0%)
Colorado	4 (15.4%)	13 (50%)	9 (34.6%)	0
Texas	10 (13.5%)	24 (32.4%)	35 (47.3%)	5 (6.8%) **
		Solo Practice		
Yes	23 (20%)	47 (40.9%)	39 (33.9%)	6 (5.2%)
No	16 (15.8%)	46 (45.5%)	35 (34.7%)	4 (4.0%)

* x^2^ = 8.05, *p* = 0.0449; ** x^2^ = 18.92, *p* = 0.0258.

**Table 3:** Physician attitude towards healthcare access and healthcare financing.

Items	Strongly Agree	Agree	Disagree	Strongly Disagree
Good medical care should be accessible to all regardless of ability to pay	108 (44.6%)	101 (41.7%)	25 (10.3%)	8 (3.3%)
I would accept a 10% reduction to income for a significant reduction in paper work	49 (20.5%)	98 (41.0%)	56 (23.4%)	36 (15.1%)
I would favor paying physician by salary (fixed annual income) if the salaries were determined in a fair way	33 (13.6%)	85 (35.1%)	73 (30.2%)	51 (21.1%)
The private insurance industry should continue to play a major role in the financing and delivery of medical care	33 (13.9%)	78 (32.8%)	69 (29.0%)	58 (24.4%)
	In Favor		Undecided	Opposed
Do you favor physicians group competing for placement in price tiered networks?	39 (16.3%)		76 (31.7%)	125 (52.1%)
Do you favor a single payer system (like England)?	84 (34.6%)		44 (18.1%)	115 (47.3%)
Do you favor the use of health savings account with a high deductible?	96 (39.7%)		71 (29.3%)	75 (31.0%)
Under a Single Payer System…	Strongly Agree	Agree	Disagree	Strongly Disagree
Emergency conditions will receive high quality care	39 (16.5%)	95 (40.1%)	63 (26.6%)	40 (16.9%)
Patients will have access to cutting edge technology	20 (8.4%)	82 (34.6%)	88 (37.1%)	47 (19.8%)
Patients will have access to preventive care	60 (25.0%)	124 (51.7%)	34 (14.2%)	22 (9.2%)
Patients health outcomes will improve	37 (15.9%)	81 (34.9%)	73 (31.5%)	41 (17.7%)
Fewer administrative staff will be necessary	39 (16.7%)	85 (36.3%)	55 (23.5%)	55 (23.5%)
Acquisition of new technology will improve	11 (4.7%)	63 (27.0%)	104 (44.6%)	54 (23.2%)
My income will decrease significantly	49 (20.9%)	94 (40.2%)	79 (33.8%)	12 (5.1%)
I will have to work longer hours	41 (17.5%)	76 (32.5%)	102 (43.6%)	15 (6.4%)
I will have more free time	12 (5.2%)	56 (24.2%)	114 (49.4%)	49 (21.2%)
I will have a fixed salary	25 (10.7%)	139 (59.4%)	62 (26.5%)	8 (3.4%)
Overall, the US economy will benefit	38 (16.2%)	67 (28.6%)	69 (29.5%)	60 (25.6%)

**Table 4:** Physician attitudes towards healthcare access and single payer system and demographic data.

Items	Strongly Agree	Agree	Disagree	Strongly Disagree
I would favor paying physician by salary (fixed annual income) if the salaries were determined in a fair way				
Female	15 (22.7%)	25 (37.9%)	18 (27.3%)	8 (12.1%)
Male	48 (23.8%)	57 (28.2%)	54 (26.7%)	43 (21.3%) *
Fewer administrative staff will be necessary under a single payer system				
Member of state medical association	20 (12.9%)	53 (26.2%)	41 (20.3%)	41 (20.3%)
Not a member of state medical association	19 (25.3%)	30 (40%)	13 (17.3%)	13 (17.3%) **
Rural primary practice location	13 (16.9%)	23 (29.9%)	25 (32.5%)	16 (20.8%)
Suburban primary practice location	6 (8.5%)	32 (45.1%)	12 (16.9%)	21 (29.6%)
Urban primary practice location	20 (24.4%)	28 (34.1%)	17 (20.7%)	17 (20.7%) ***
I will have a fixed salary under a single payer system				
Solo practitioner	9 (7.7%)	75 (64.1%)	25 (2.4%)	8 (6.8%)
Not a solo practitioner	15 (14.0%)	60 (56.1%)	32 (29.9%)	0 (0%) †
Overall, the US economy will improve under a single payer system				
Member of state medical association	17 (11.0%)	44 (28.4%)	52 (33.5%)	42 (27.1%)
Not a member of state medical association	20 (26.3%)	23 (30.3%)	16 (21.1%)	17 (22.4%) ††
	In Favor		Undecided	Opposed
Do you favor a single payer system (like England)?				
New York	48 (43.2%)		16 (14.4%)	47 (42.3%)
Texas	17 (21.3%)		14 (17.5%)	49 (61.3%)
Mississippi	5 (41.7%)		3 (25%)	4 (33.3%)
Colorado	9 (30%)		10 (33.3%)	11 (36.7%) †††
Member of state medical association	47 (29.4%)		28 (17.5%)	85 (53.1%)
Not a member of state medical association	36 (46.2%)		14 (17.9%)	28 (35.9%) §
Rural primary practice location	32 (41.0%)		23 (29.5%)	23 (29.5%)
Suburban primary practice location	37 (50%)		24 (32.4%)	13 (17.6%)
Urban primary practice location	26 (30.2%)		22 (25.6%)	38 (44.2%) §§
Do you favor the use of health savings account with a high deductible?				
Member of state medical association	69 (43.4%)		54 (34.0%)	36 (22.6%)
Not a member of state medical association	25 (32.1%)		15 (19.2%)	38 (48.7%) §§§

* x^2^ = 9.44, *p* = 0.0239; ** x^2^ = 8.66, *p* = 0.0342; *** x^2^ = 14.05, *p* = 0.0291; † x^2^ = 11.6, *p* = 0.0089; †† x^2^ = 10.71, *p* = 0.0134; ††† x^2^ = 16.92, *p* = 0.0096; § x^2^ = 7.52, *p* = 0.0233; §§ x^2^ = 13.74, *p* = 0.0082; §§§ x^2^ = 16.99, *p* = 0.0002.

**Table 5:** Patient-practitioner orientation scale (PPOS).

Elements	Strongly Agree (*n*, %)	Agree (*n*, %)	Disagree (*n*, %)	Strongly Disagree (*n*, %)
If physicians are good at diagnosis and treatment, the way they relate to patients is not as important.	4 (1.6%)	9 (3.7%)	87 (35.7%)	143 (58.6%)
Patients should be treated as partners with the physician, equal in power and status regarding health decisions *	88 (35.9%)	123 (50.2%)	27 (11.0%)	6 (2.4%)
Patients generally want reassurance rather than information about their health	7 (2.9%)	75 (31.3%)	127 (52.9%)	31 (12.9%)
Clinical disagreements between the physician and the patient, is a sign that the physician does not have the patient’s trust	8 (3.3%)	72 (29.6%)	135 (55.6%)	28 (11.5%)
A treatment plan cannot succeed if it is in conflict with a patient’s lifestyle or value *	76 (30.9%)	140 (56.9%)	28 (11.4%)	2 (0.8%)
Most patients want to get in and out of the physician’s office as quickly as possible	35 (14.3%)	94 (38.5%)	92 (37.7%)	23 (9.4%)
Humor is a factor in the physician’s treatment of the patient *	64 (26.6%)	148 (61.4%)	22 (9.1%)	7 (2.9%)
Patients should know about their physician’s financial relationships with drug and medical device companies	63 (26.1%)	104 (43.2%)	50 (20.7%)	23 (9.5%)

* Reverse coded.

**Table 6:** Patient-practitioner orientation factor loadings.

	Physician-Patient Relationship	Physician-Patient Engagement	Patient Conflict
Patients should be treated as partners with the physician, equal in power and status regarding health decisions *	0.69		
Humor is a factor in the physician’s treatment of the patient *	0.61		
Patients should know about their physician’s financial relationships with drug and medical device companies	−0.67		
If physicians are good at diagnosis and treatment, the way they relate to patients is not as important.		0.72	
Most patients want to get in and out of the physician’s office as quickly as possible		0.63	
Patients generally want reassurance rather than information about their health		0.59	
Clinical disagreements between the physician and the patient, is a sign that the physician does not have the patient’s trust			0.78
A treatment plan cannot succeed if it is in conflict with a patient’s lifestyle or value * §			

* Reverse coding; § This statement loaded on multiple factors.

**Table 7:** Four different healthcare financing structures and PPOS.

	Multi-Payer Managed Care System	Single Payer with Universal Coverage	Individualized Insurance Coverage (Using Health Savings Account)	Employer Sponsored Healthcare System with Tax Credit or Penalty (For Buying or not Buying Health Insurance)
Perceived Weaker Physician-Patient Relationship	10 (50%)	0 (0%)	10 (50%)	0 (0%)
Perceived Neutral Physician-Patient Relationship	21 (13.9%)	65 (43%)	55 (36.4%)	4 (6.6%)
Perceived Stronger Physician-Patient Relationship	4 (9.3%)	29 (67.4%)	8 (18.6%)	2 (4.7%)

x^2^ = 36.146, *p* < 0.0000001.

**Table 8:** Factor loadings—single payer system.

	System Orientation	Individual Orientation
Emergency conditions will receive high quality care	0.825	
Acquisition of new technology will improve	0.844	
Patients will have access to cutting edge technology	0.881	
Patients will have access to preventive care	0.806	
Patients health outcomes will improve	0.894	
Fewer administrative staff will be necessary	0.749	
My income will decrease significantly		0.811
I will have to work longer hours		0.744
I will have a fixed salary		0.748
Overall, the US economy will benefit	0.81	

One question (I will have more free time) loaded on both factors without showing significant loading either.

**Table 9:** Single payer system and patient-practitioner orientation.

	Less System Orientation	Neutral System Orientation	More System Orientation	Less Individual Orientation	Neutral Individual Orientation	More Individual Orientation
Physician perception of physician-patient relationship						
Weaker	6 (16.7%)	22 (61.1%)	8(22.2%)	9 (25%)	19(52.8%)	8 (22.2%)
Neutral	4 (3.3%)	103 (83.7%)	16 (13%)	23 (15.9%)	102 (70.3%)	20 (13.8%)
Stronger	0 (0%)	5 (41.7%)	7 (58.3%) *	0 (0%)	22 (75.9%)	7 (24.1%) **
Physician perception of patient conflict						
Less conflict	7 (20.6%)	17 (50%)	10 (29.4%)	9 (26.5%)	15 (44.1%)	10 (29.4%)
Neutral conflict	18 (13.4%)	97 (72.4%)	19 (14.2%)	12 (9%)	103 (76.9%)	19 (14.2%)
More conflict	14 (32.6%)	27 (62.8%)	2 (4.7%) ***	11 (26.2%)	25 (59.5%)	6 (14.3%) ****

* x^2^ = 26.015, *p* < 0.000001; ** x^2^ = 10.669, *p* = 0.031; *** x^2^ = 16.694, *p* = 0.002; **** x^2^ = 18.384, *p* = 0.001.
